# Analysis of fracture healing in osteopenic bone caused by disuse: experimental study

**DOI:** 10.1590/1414-431X20155076

**Published:** 2016-02-02

**Authors:** A.G. Paiva, G.R. Yanagihara, A.P. Macedo, J. Ramos, J.P.M. Issa, A.C. Shimano

**Affiliations:** 1 Departamento de Biomecânica, Medicina e Reabilitação do Aparelho Locomotor, Faculdade de Medicina de Ribeirão Preto, Universidade de São Paulo, Ribeirão Preto, SP, Brasil; 2 Departamento de Morfologia, Fisiologia e Patologia Básica, Faculdade de Odontologia de Ribeirão Preto, Universidade de São Paulo, Ribeirão Preto, SP, Brasil

**Keywords:** Osteoporosis, Osteotomy, Fracture healing, Immobilization, Rats

## Abstract

Osteoporosis has become a serious global public health issue. Hence, osteoporotic fracture healing has been investigated in several previous studies because there is still controversy over the effect osteoporosis has on the healing process. The current study aimed to analyze two different periods of bone healing in normal and osteopenic rats. Sixty, 7-week-old female Wistar rats were randomly divided into four groups: unrestricted and immobilized for 2 weeks after osteotomy (OU2), suspended and immobilized for 2 weeks after osteotomy (OS2), unrestricted and immobilized for 6 weeks after osteotomy (OU6), and suspended and immobilized for 6 weeks after osteotomy (OS6). Osteotomy was performed in the middle third of the right tibia 21 days after tail suspension, when the osteopenic condition was already set. The fractured limb was then immobilized by orthosis. Tibias were collected 2 and 6 weeks after osteotomy, and were analyzed by bone densitometry, mechanical testing, and histomorphometry. Bone mineral density values from bony calluses were significantly lower in the 2-week post-osteotomy groups compared with the 6-week post-osteotomy groups (multivariate general linear model analysis, P<0.000). Similarly, the mechanical properties showed that animals had stronger bones 6 weeks after osteotomy compared with 2 weeks after osteotomy (multivariate general linear model analysis, P<0.000). Histomorphometry indicated gradual bone healing. Results showed that osteopenia did not influence the bone healing process, and that time was an independent determinant factor regardless of whether the fracture was osteopenic. This suggests that the body is able to compensate for the negative effects of suspension.

## Introduction

Osteoporosis is one of the most prevalent bone diseases and it represents a serious public health problem. Osteoporosis is characterized by low bone mineral density (BMD) and microarchitecture deterioration that causes fragility, making bone fractures its main clinical consequence (1 2 3). Osteoporotic fractures represent a considerable risk of reduced quality of life or mortality, leading to high medical costs (4).

Osteoporosis affects about 200 million people worldwide (5). In the United States, about 10 million people aged over 50 years have osteoporosis, and another 34 million have osteopenia (6). The osteoporotic fracture rate in Brazil is 15% for women and 13% for men over 40 years old (7). Because osteoporosis can cause bone fractures even after mild trauma (8), an individual with a low fracture threshold is highly likely to sustain a fracture merely by falling from his/her own height (9).

Previous studies on osteoporotic fracture healing have demonstrated the influence of osteoporosis in both the initial (10) and late (11) bone healing phases. Osteoporosis can speed up the initial healing phase and delay bony callus mineralization that forms between and around the edges of bone fractures (12), and speeds up the fracture-healing processes (13).

Although recent studies have shown effects of bone microarchitecture loss on the bone healing process (14), others have shown that such a link does not exist (15). Despite advances in studies on bone healing, the results are controversial and the relationship between healing time and the osteopenic condition remains unclear. Thus, this study aims to evaluate the influence of osteopenia on bone healing time in an experimental model of partial fracture after trauma.

## Material and Methods

This experimental protocol was approved by the Ethics Committee on Animal Experimentation of Faculdade de Medicina de Ribeirão Preto, Universidade de São Paulo (FMRP, USP; protocol #003/2013). The procedures performed in the study were consistent with standards described by the International Guiding Principles for Biomedical Research Involving Animals (16), and by the Brazilian College of Animal Experimentation.

Sixty, 7-week-old female Wistar rats were acquired from the Bioterium of Ribeirão Preto Campus, USP. The animals were randomly divided into four experimental groups (n=15; Table 1). Thirty female rats were subjected to the tail suspension method to induce osteopenia (17,18); they remained suspended for 21 days according to recommendations given by Morey-Holton and Globus (19). The other 30 rats remained unrestricted for the same period of time (21 days). All animals were provided with water and food *ad libitum* and were housed in individual cages specifically developed for the suspension system in a controlled temperature room (21±2°C) with a 12h light/dark cycle. The room was located in the Bioterium of the Bioengineering Laboratory at FMRP, USP.

**Table:**
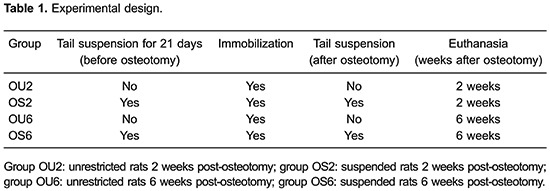

After 21 days, which was the period stipulated for osteopenia to establish itself in the suspended animals, all animals were anesthetized with a combination of 0.6 mL/kg ketamine hydrochloride and 0.4 mL/kg xylazine to allow the osteotomy to be performed.

**Figure 1:**
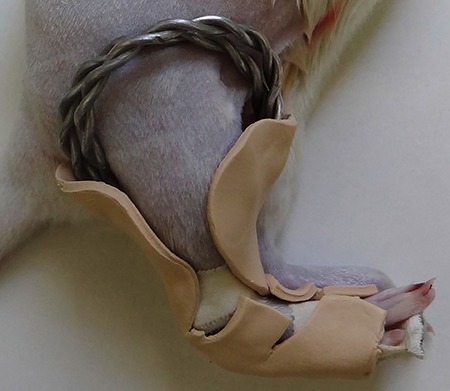
Fractured right hindlimb immobilized by an orthosis.

Partial osteotomy was performed on the right tibial diaphysis, with animals in the prone position with external rotation of the hip and triple flexion. A longitudinal skin incision of approximately 1 cm was made, and then muscle spacing was performed to expose the medial bone surface of the tibia. Partial osteotomy of approximately 2.7 mm profundity was performed in the right tibial diaphysis with bone lateral face preservation using a low rpm engine (Micro Motor 210/105L, Strong¯, Korea) and a cutting cylindrical drill (PM 699, Jet¯ Carbide Burs, Switzerland). After suturing, the limb was immobilized with an orthosis specifically developed for this work (Figure 1) based on the modified Thomas apparatus (20). The use of the orthosis was required to ensure proper bony callus formation, which is responsible for the regeneration of bone in the fractured region. Postoperative assessment was performed daily until the end of the experiment, and orthoses were replaced when necessary.

After the experimental period, rats were euthanized with excess anesthetic (ketamine hydrochloride and xylazine), and the right (osteotomy) and left (internal control) tibias were dissected and wrapped in gauze soaked in saline solution (n=10) and stored at -20°C. Tibias were thawed for 24 h in a refrigerator at 4°C, and for 2 h at room temperature before conducting the analyses (bone densitometry and mechanical testing). The right tibias (n=5) were immediately fixed in 4% paraformaldehyde for histological analysis.

### Bone mineral density

The BMD of the bony callus region of interest (ROI), and the left tibias (total) was measured with a high resolution dual-energy X-ray absorptiometry (DEXA) densitometer (Discovery™ Hologic QDR¯ Series, USA), with the QDR software especially designed for small animals. In all analyses, the ROI was a square with the same dimensions positioned in the middle third of the tibia.

### Mechanical testing

**Figure 2:**
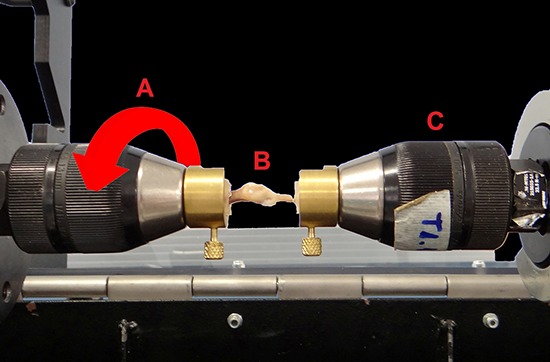
Positioning of the tibia to test the torsion. *A*: mobile device, arrow indicates the orientation of the torsion; *B*: tibia; *C*: fixed device.

Low speed mechanical torsion testing was performed using an INSTRON¯ 55MT (USA) after images were acquired by DEXA. The ends of the tibias were embedded in acrylic resin. This embedding, together with the use of a specific accessory, allowed the correct positioning of the tibia in the machine (Figure 2). A 22-Nm load cell was used, and torsion was performed at a speed of 10°/min in a counterclockwise direction. The distal portion remained fixed and the proximal portion was movable. Maximum torque, angle at maximum torque, and stiffness values were obtained.

### Histomorphometric analysis

Samples were fixed in 4% formaldehyde, decalcified in 10% ethylenediamine tetraacetic acid, dehydrated, diaphanized and embedded in paraffin. Serial sections (5 μm) were then taken along the sagittal plane of the tibia. The slides were stained with Masson's Trichrome and Picro-Sirus Red to quantify the neoformation of bone and type I and III collagen fibers, respectively. Images were obtained through an Axion Imager Z2 optical microscope (Zeiss¯, Germany) coupled to a digital camera (Zeiss). Histomorphometric analysis was performed on the bony callus only (right tibia). A test system was used to differentiate type I and III collagen (identified by different staining color).

### Statistical analyses

Data are reported as means±SD. Statistical analyses were performed using SPSS for Windows version 17.0 (SPSS, USA). Multivariate analysis was performed with a general linear model (random and fixed effects) for data analysis in which responses obtained from the same animal were grouped. Assumptions on the independence among observations performed in the same group were not appropriate. Bonferroni's correction was performed for multiple comparisons. P≤0.05 was considered to be statistically significant.

## Results

Results for the tibias that underwent osteotomy are shown in Table 2 and the internal control results are listed in Table 3. After dissecting the right tibia, 55% of the bony calluses in the 2-week post-osteotomy groups showed incomplete healing and exuberant bony calluses.

**Table:**
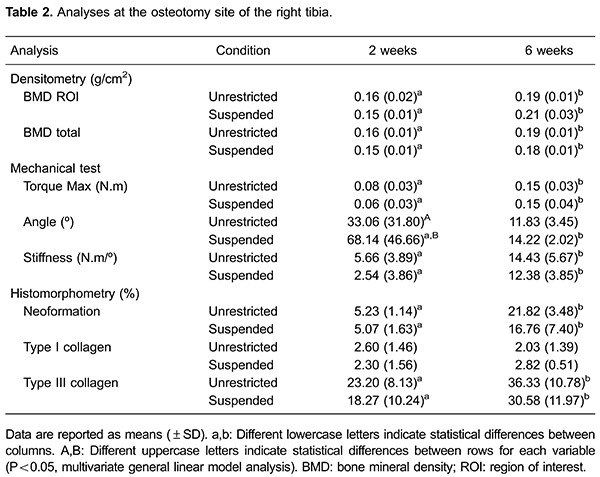

**Table:**
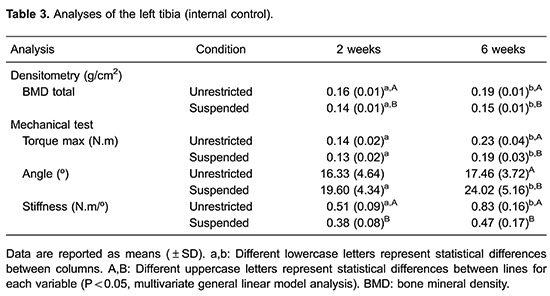

### Bone mineral density

The BMD of the bony calluses was significantly higher after 6 weeks of healing time compared with 2 weeks (P<0.000); however, suspension and the time × suspension interaction had no significant effect (P>0.05) on BMD. The BMD of the OS2 group was significantly lower than that of the OS6 group (P<0.000). Similarly, the BMD of the OU2 group was significantly lower than that of the OU6 group (P<0.05).

### Mechanical testing

The maximum torque and stiffness of the right tibias was significantly higher after 6 weeks of healing time compared with 2 weeks (P<0.000); however, suspension and the time × suspension interaction had no significant effect (P>0.05) on maximum torque and stiffness. Maximum torque and stiffness were significantly lower in the OS2 group compared with the OS6 group (P<0.000); significantly lower values were also found in the OU2 group compared with the OU6 group (P<0.000).

The angle at maximum torque of the right tibias was significantly lower after 6 weeks healing time compared with 2 weeks (P<0.000), whereas suspension and the time × suspension interaction had no significant effect (P>0.05). Significantly larger angles at maximum torque were observed in the OS2 group compared with the OS6 group (P<0.000) and OU2 group (P<0.02).

### Histomorphometric analysis

**Figure 3:**
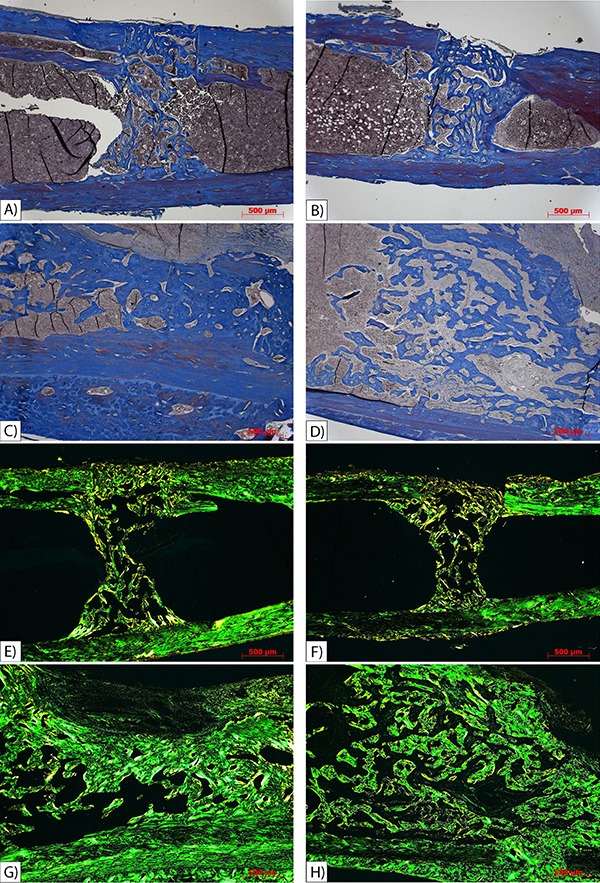
Histological photomicrography of tibia sections stained by Trichrome Masson (*A-D*) and Picro-Sirus Red (*E-H*), original magnification 50×. Group OU2 (*A*) and group OS2 (*B*) showed lower amounts of bone formation than group OU6 (*C*) and group OS6 (*D*). Group OU2 (*E*) and group OS2 (*F*) showed lower collagen levels than group OU6 (*G*) and group OS6 (*H*). Group OU2: unrestricted rats 2 weeks post-osteotomy; group OS2: suspended rats 2 weeks post-osteotomy; group OU6: unrestricted rats 6 weeks post-osteotomy; group OS6: suspended rats 6 weeks post-osteotomy.

There was significantly greater bone neoformation after 6 weeks healing time compared with 2 weeks (P<0.000), whereas suspension and the time × suspension interaction had no significant effect (P>0.05). Significantly lower bone neoformation was observed in the OS2 group compared with the OS6 group (P<0.000), and it was also significantly lower in the OU2 group compared with the OU6 group (P<0.000; Figure 3).

The amount of type I collagen was not significantly different according to bone healing time (P=0.959), suspension (P=0.683) or the time × suspension interaction (P=0.357). There was a significantly greater amount of type III collagen after 6 weeks healing time compared with 2 weeks (P=0.014); however, suspension (P=0.267) and the time × suspension interaction (P=0.931) had no significant effect on the amount of type III collagen. A significantly smaller amount of collagen was found in the OS2 group compared with the OS6 group (P<0.05), and the OU2 group had a significantly lower amount of collagen compared with the OU6 group (P<0.05).

## Discussion

The difference in bone healing between osteopenic and healthy bones remains unclear. The current study investigated bone healing times in rats with osteopenia due to immobilization, as a model for the commonly occurring frame fractures in patients suffering from bone metabolic disorders and old age. Length of healing time was found to be the main determinant for bone healing, and the presence of osteopenia did not interfere in the healing process. It is suggested that the body is able to counterbalance the possible negative effects of suspension through biological adaptation and compensatory mechanisms for fracture healing.

Several previous studies have investigated the influence of osteoporosis on bone healing using ovariectomized rats as an experimental model; this is a well-studied method that simulates the changes in postmenopausal women, and needs an average of 3 months for the osteopenic condition to onset (1,3,10,13 14 15,). Our study differed from these previous studies in that the osteopenic condition was achieved by the tail suspension method; this method simulates disuse, and osteopenia occurs in only 21 days. The animals were kept suspended until the end of the experiment, with no discharge during recovery. The effectiveness of the suspension method in inducing osteopenia is well described in the literature (17,18,24,25), and has been confirmed via comparison with internal controls in the current study (Table 3). In osteopenia, the cortical bone (diaphysis) is less affected than the trabecular bone (metaphysis) (23). Our mechanical analysis of the left tibias showed that the cortical bone was weaker in the suspended animals, confirming the mechanical results obtained by Bloomfield et al. (17).

There are two methods for treating fractures: conservative (using a plaster cast, splint, and orthosis) and surgical (using plates, screws, Kirschner wire, and percutaneous pins) (7,26). Immobilization is crucial for bone healing because a minimum cortical contact is required for the bone to join properly (7); based on this principle, the best treatment method can be chosen according to each fracture’s particular characteristics. In the current study, we opted for conservative treatment (orthosis) because only partial osteotomy was performed. The results were satisfactory, and orthosis did not influence the bone healing process in any of the experimental groups. This result is similar to a previous study using total tibial osteotomy in which surgical intramedullary nailing used for immobilization did not interfere in the bone healing process (15).

The BMD and mechanical properties of the bony calluses in the unrestricted (normal) animals were not different from that in the calluses of the suspended (osteopenic) animals. This corroborates previous findings using the ovariectomy (OVX)-induced osteopenic model (10,15). Differences between normal and osteopenic animals were found only in bone healing time, similar to previous findings (21 [Bibr B22]). Such results can be explained by the bone healing process; the bony callus is still in the immature stage at 2 weeks, while the 6-week-old bony callus shows increased mineral deposition.

At the end of the experiment, there was a high rate of incomplete healing and exuberant bony calluses in both the suspended and unrestricted 2-week post-osteotomy groups, owing to poor bone neoformation in groups with shorter bone healing time. This is similar to a previous study in which the amount of collagen and cartilage exceeded the amount of immature bone tissue in the first 2 weeks of bone healing (27). Type III collagen develops in response to growth factors for the growth and healing of fractures (28). We observed a higher amount of type III collagen in the 6-week post-osteotomy groups, representing more mature bone healing, compared with the 2-week post-osteotomy groups.

There were no significant changes in the mechanical properties of the bony callus in the unrestricted rats compared with those in the suspended rats; this was supported by the densitometry and histology results. This is in accordance with a previous study that also found no difference in OVX animals compared with normal animals (15). However, bone healing time changed the mechanical properties of the bony calluses, regardless of whether the bones were osteopenic, as evidenced by the significant difference in maximum torque and stiffness between the groups 2 and 6 weeks after osteotomy. Changes such as lower BMD and larger callus size may have contributed to the decrease in mechanical properties found in the 2-week post-osteotomy groups because a larger bony callus also has a larger cross-sectional area, which directly influences material torsional strength.

The healing process starts as soon as a fracture occurs; cartilage and bone tissue are formed to stabilize the fracture. The histological sections revealed a smaller amount of newly formed bone 2 weeks post-osteotomy compared with 6 weeks post-osteotomy, regardless of the presence of osteopenia. As the callus matures, the new bone tissue is remodeled until it achieves bone maturity. As callus maturation progresses, mechanical integrity and functional qualities return to normal bone conditions (13).

In the current study, fracture repair was found to be a regulated process orchestrated to restore the structural geometry, mechanical properties and mobility of the fractured bone, as previously described (13). New bone replaces the collagen and cartilage between the fifth and sixth week of bone healing (27); that is, the amount of mineral deposition increases gradually as a function of time. Hence the BMD, maximum torque and stiffness values of the bony callus in the 2-week post-osteotomy groups were significantly lower than in the 6-week post-osteotomy groups.

There were some limitations of the present study. First, we examined the bone healing only in young rats. There could potentially be a difference in bone healing in immobilized versus unrestricted aged rats because aging slows down bone metabolism. Second, we developed the osteopenic model using the suspension method, and the changes obtained by this method are not systemic, unlike the changes obtained when OVX is performed. However, the purpose of this was to simulate disuse. This study provides a basis for future work in this area.

In conclusion, our experimental model showed that time was the key factor for healthy bone healing, and the presence of osteopenia did not interfere in the fracture healing process.
